# The Impact of Culture Medium on Morphokinetics of Cleavage Stage Embryos: An Observational Study

**DOI:** 10.1007/s43032-022-00962-7

**Published:** 2022-05-09

**Authors:** Linette van Duijn, Melek Rousian, Charlotte S. Kramer, Eva S. van Marion, Sten P. Willemsen, Jeroen P. Speksnijder, Joop S. E. Laven, Régine P. M. Steegers-Theunissen, Esther B. Baart

**Affiliations:** 1 Department of Obstetrics and Gynecology, Erasmus MC, University Medical Center Rotterdam, Rotterdam, The Netherlands; 2 Division of Human Nutrition and Health, Wageningen University and Research, Wageningen, The Netherlands; 3 Division of Reproductive Endocrinology and Infertility, Department of Obstetrics and Gynecology, Erasmus MC, University Medical Center Rotterdam, Dr. Molewaterplein 40, Rotterdam, 3015 GD The Netherlands; 4 Department of Biostatistics, Erasmus MC, University Medical Center Rotterdam, Rotterdam, The Netherlands; 5 Department of Developmental Biology, Erasmus MC, University Medical Center Rotterdam, Rotterdam, The Netherlands

**Keywords:** Time-lapse imaging, Culture medium, Embryo development, Morphokinetics, Preimplantation

## Abstract

To study the impact of culture media on preimplantation morphokinetics used for predicting clinical outcomes. All IVF and ICSI cycles performed between 2012 and 2017 with time-lapse information available were included. In November 2014, culture medium was changed from Vitrolife G-1 PLUS to SAGE 1-Step. Each embryo was retrospectively assigned a morphokinetic-based KIDScore for prediction of implantation. Clinical outcomes were retrieved from medical records. Linear mixed models were used to study differences in morphokinetic parameters, a proportional odds model for KIDScore ranking and logistic regression for differences in clinical outcomes. All analyses were adjusted for patient and treatment characteristics. In 253 (63.1%) cycles, embryos (*n* = 671) were cultured in Vitrolife, and in 148 (36.9%) cycles, embryos (*n* = 517) were cultured in SAGE. All cleavage divisions occurred earlier for SAGE embryos than for Vitrolife embryos (2-cell: -2.28 (95%CI: -3.66, -0.89), 3-cell: -2.34 (95%CI: -4.00, -0.64), 4-cell: -2.41 (95%CI: -4.11, -0.71), 5-cell: -2.54 (95%CI: -4.90, -0.18), 6-cell: -3.58 (95%CI: -6.08, -1.08), 7-cell: -5.62 (95%CI: -8.80, -2.45) and 8-cell: -5.32 (95%CI: -9.21, -1.42) hours, respectively). Significantly more embryos cultured in SAGE classified for the highest KIDScore compared to embryos cultured in Vitrolife (*p* < 0.001). No differences were observed in clinical outcomes. Our results demonstrate an impact of culture medium on preimplantation embryo developmental kinetics, which affects classification within the KIDScore algorithm, while pregnancy outcomes were comparable between the groups. This study underscores the need to include the type of culture medium in the development of morphokinetic-based embryo selection tools.

**Supplementary Information:**

The online version contains supplementary material available at 10.1007/s43032-022-00962-7.

## Introduction

Since the first successful in vitro fertilisation (IVF) treatment in 1978, delivery rates have increased to over 30% per oocyte pickup [1–4]. This increase can be attributed to several advances, which have led to improvements in culture conditions [5–7]. However, in vitro culture conditions still poorly mimic the human in vivo environment and it has been hypothesised that the in vitro environment during IVF exposes the preimplantation embryo to additional stressors. To minimise the detrimental effects of the in vitro culture environment on human embryo development, past research aimed to improve culture media for IVF. Two competing approaches regarding IVF media have been advocated, often paraphrased as: ‘*back to nature’* and ‘*let the embryo choose’* [8].

The first approach is based on the knowledge of embryo metabolism and compositions of fluids in the female reproductive tract, resulting in sequential media for fertilisation, the cleavage stages and blastocyst development [9]. For the second approach, culture media components were systematically adjusted using mouse preimplantation embryos, resulting in a single-step formulation that enabled culture of human embryos from fertilisation up to the blastocyst stage [10–12].

Although studies suggest that culture in single-step media results in the development of more blastocysts than culture in sequential culture media, clinical outcomes are similar [13–16]. This may be attributed to either differences in the composition of media or to the fact that single-step culture media allow undisturbed embryo culture, as there is no need to transfer the embryo to the next culture medium on day 3 of development [17]. Despite comparable implantation rates between media, some studies report differences in foetal size, birthweight and even postnatal weight at 2 years [18–20]. Recently we have demonstrated that these differences can arise as early as the first trimester, as embryos cultured in SAGE 1-Step exhibit faster growth and development than embryos cultured in Vitrolife G-1 PLUS [21].

Little is known whether these differences are also observed prior to implantation. Since 1997, preimplantation embryo development can be closely observed with time-lapse imaging [22]. This technique is increasingly used to investigate the association between preimplantation development and implantation, and to improve embryo selection for transfer. However, prospective randomised trials on time-lapse-based embryo selection report conflicting results on the improvement of success rates and indicate that embryo developmental kinetics are subject to patient related factors and local laboratory variables [23–25]. To prevent that these variables affect embryo selection, an universally applicable algorithm for embryo selection has been developed [26]. This algorithm, the Known Implantation Data (KID) Score, is a (de)selection tool that ranks embryos according to their implantation potential, regardless of patient characteristics, the fertilisation technique used and culture conditions applied. Moreover, it has a high blastulation predictability and performs superior to conventional morphology evaluation for predicting live births, when applied to day 3 embryos [27].

Reports on the impact of the type of culture medium on morphokinetics are conflicting, and the impact on the performance of morphokinetic selection algorithms remains poorly investigated [28–31]. In this study, we retrospectively compare morphokinetics between embryos cultured in Vitrolife G-1 PLUS™ and SAGE 1-Step™. The primary aim of this study is to investigate associations between these two culture media and preimplantation embryo morphokinetic parameters in both IVF and intracytoplasmic sperm injection (ICSI) treatments. The secondary aim is to investigate the impact of culture medium on the predicted implantation potential as assessed by the KIDScore algorithm. This is compared to the observed implantation potential by studying the associations between culture media and clinical outcomes. Expanding our knowledge will not only help in understanding the role of culture medium in preimplantation embryonic development, but can also help in improving embryo selection.

## Methods

### Study design and participants

This patient registry cohort study was conducted between 31 January 2012 and 26 July 2017 at the outpatient Fertility Clinic of the Division of Reproductive Endocrinology and Infertility, Department of Obstetrics and Gynaecology of the Erasmus MC, University Medical Center Rotterdam, the Netherlands. Couples undergoing IVF or ICSI treatment with ejaculated sperm, who had their embryos cultured in the EmbryoScope™ time-lapse incubator (Vitrolife, Goteborg, Sweden), were included. If a couple underwent multiple cycles during the study period, only the first treatment cycle was included. This is defined as the first cycle for the wish to conceive during the study period. The morphokinetic parameters were extracted from the EmbryoViewer™ database, while data on patient characteristics and clinical outcomes were retrieved from medical records. A (post hoc) sample size calculation revealed that a sample of at least 132 embryos per culture medium had 80% power to detect a 1-h difference with a significance level (α) of 5% (two-tailed).

### Ovarian stimulation, oocyte retrieval and IVF procedures

Ovarian stimulation, oocyte retrieval and IVF and ICSI procedures were performed as described previously, with the following modifications: women underwent ovarian stimulation with either a GnRH agonist or GnRH antagonist combined with recombinant follicle-stimulating hormone (rFSH) or urinary FSH administration [32, 33]. Ovarian stimulation protocols are standardised at our centre, and the distribution of GnRH agonist or GnRH antagonist protocols reflects policy changes over time and not patient selection. FSH or rFSH dosage was based on female age, antral follicle count and prior response to gonadotrophins (if applicable). Final follicular maturation, i.e. ovulation, was triggered with human chorionic gonadotrophin (hCG) or a GnRH agonist. Oocytes were collected 35 h later and cultured in G-IVF PLUS (Vitrolife, Goteborg, Sweden) during the period from January 31, 2012, to November 17, 2014. From November 17, 2014, onwards, oocytes were cultured in SAGE human tubal fluid medium (HTF, CooperSurgical, Trumbull, CT, USA) and supplemented with 5% human serum albumin (CooperSurgical) under an oil overlay (CooperSurgical). Oocytes were subsequently fertilised conform routine IVF or ICSI procedures. For manipulation of the oocytes for ICSI outside the incubator, either G-MOPS plus (Vitrolife) or SAGE HEPES-buffered HTF (CooperSurgical) was used.

### Embryo culture, annotations and transfer

IVF oocytes were cultured overnight in drops of 100 µl fertilisation medium in universal GPS dishes (CooperSurgical) under oil after insemination. The next morning, only fertilised oocytes with two pronuclei were transferred to an EmbryoSlide (Vitrolife). Denuded ICSI oocytes were transferred to an EmbryoSlide directly after injection. Oocytes or embryos were individually cultured in the EmbryoScope in 25 µl of culture medium under 1.4 ml oil.

Between January 31, 2012, and November 17, 2014, embryos were cultured using G-1 PLUS medium (Vitrolife). After embryo selection for transfer on day 3, remaining embryos were transferred to a fresh EmbryoSlide containing G-2 PLUS medium. From November 17, 2014, onwards, SAGE 1-Step medium (Cooper Surgical) was used for culture from day 0/1 until day 4, primarily for practical reasons. During the complete study period, fresh embryo transfer (ET) was performed on day 3 after ovum pickup. Embryos were evaluated on a single image acquired 66–68 h after fertilisation without support of time-lapse information and selection was based on number of blastomeres, fragmentation, size equality and signs of early compaction. Remaining embryos with more than 12 blastomeres or 30% of compaction were selected for cryopreservation on day 4.

Embryos were cultured at 36.8 °C in an atmosphere containing 7% oxygen. To achieve a stable pH level of 7.2–7.3, the level of CO_2_ was adjusted to 5.5% for the Vitrolife media and 4.5% CO_2_ for the SAGE media in all incubators. In our clinic, it is standard care to transfer a single embryo. Only women aged ≥ 38 years without medical contraindications or women who underwent 2 or more fresh IVF or ICSI cycles could opt for double embryo transfer. Pregnancy was confirmed biochemically by a positive hCG test (two weeks after ET) and by ultrasound (5 weeks after ET). The cumulative pregnancy rate was defined as a pregnancy resulting from either fresh ET or any frozen–thawed ET of embryos from the treatment cycle included in the study cohort within a two-year follow-up period.

### Time-lapse imaging and assessment

Every 10 minutes, images were recorded automatically in seven focal planes (15 μm intervals) with a monochrome CCD camera after exposure to a single red LED (635 nm, < 0.1 s per image, total light exposure time < 50 s/day per embryo). For IVF embryos, t = 0 was defined as the moment of insemination. For ICSI embryos, t = 0 was defined as the moment of injection of the last oocyte. Depending on the number of oocytes, the total procedure takes between 20–50 min. Manual annotations were performed according to the definitions and guidelines of the ESHRE consensus for dynamic monitoring of human preimplantation development [34]. All freshly transferred and cryopreserved embryos were individually annotated for the following developmental time points: tPNf, t2, t3, t4, t5, t6, t7 and t8. tPNF was defined as the first frame on which both pronuclei had faded. The exact timings of reaching the 2-, 3-, 4-, 5-, 6-, 7- and 8-cell stage of each embryo were defined as t2, t3, t4, t5, t6, t7 and t8, respectively. Furthermore, intervals between developmental timings were calculated (t3-t2 and t5-t4). If the interval t3-t2 was 5 h or less, the embryo was registered as direct unequal cleaving (DUC). These parameters were used by the Vitrolife® embryo-viewer software to assign each embryo a KIDScore. This score is based on the KIDScore algorithm (Vitrolife), a generally applicable morphokinetic algorithm for implantation based on six parameters (Supplemental Table 1) [26]. In this algorithm, a KIDScore of 1 corresponds with a low predicted implantation potential, whereas embryos classified as score 5 have a high predicted implantation potential (5% vs. 36% in the dataset based on which the model was developed). The interobserver and intraobserver agreement for developmental events observed by time-lapse images is reported to be high [35–37]. Internal validation of interobserver reproducibility demonstrated extremely close agreement for the timings of tPNf until t5 (intraclass correlation coefficient (ICC) > 0.95). A moderate agreement was found for t6, t7 and t8 (ICC 0.23–0.40).

### Statistical analyses

Continuous baseline data were tested using Mann–Whitney U tests, and categorical baseline data were tested using Chi-square tests. Time-lapse data were analysed using linear mixed models, which offer the possibility to analyse repeated measurements, multiple embryos from the same woman and are able to deal with missing values. In these models, time-lapse parameters were the response variables and culture medium was the independent variable. To adjust for potential confounders, two models were constructed. Model 1 only took similarities between embryos from the same women, also known as clustering, into account. Model 2 was additionally adjusted for female age, fertilisation method (IVF or ICSI), type of ovarian stimulation protocol (agonist or antagonist), culture under low oxygen conditions and the passage of time. The latter variable is based on the assumption that overall embryo quality gradually improves over time due to minor and unknown improvements in practice [38–40]. Two sub-analyses regarding morphokinetic development were performed. The first was performed in ICSI treatments only, as t = 0 corresponds to the actual moment of fertilisation in ICSI embryos, whereas in IVF embryos there is an approximately 2-h delay between insemination and fertilisation [41]. For this analysis, similar models were constructed but not adjusted for fertilisation method. The second sub-analysis was performed in freshly transferred embryos that implanted successfully, using linear regression, as only one embryo per patient is considered and there is no effect of clustering.

The effect of the culture medium on the KIDScore was investigated using a proportional odds model, a model for ordinal outcomes with patient specific intercepts to account for correlation between embryos [42]. Here, we also performed a sub-analysis that considered only the group of freshly transferred embryos that implanted successfully, by using a Chi-square test.

Clinical outcomes were analysed using logistic regression and adjusted for female age, fertilisation method and stimulation protocol, as other laboratory procedures were similar between the two media.

Embryos cultured in Vitrolife were used as reference in all analyses. All statistical analyses were performed in SPSS statistics 24.0 (IBM, Armonk, USA) and R (R: A language and Environment for Statistical Computing, version 3.1.3, 2015 for Windows, R Core Team, Vienna, Austria). Two-sided p-values < 0.05 were considered significant.

## Results

Four hundred and one treatment cycles were included in this study population. In 253 (63.1%) cycles, the embryos (*n* = 671) were cultured in Vitrolife medium; in the remaining cycles (*n* = 148, 36.9%), embryos (*n* = 517) were cultured in SAGE medium. This number of embryos is sufficient to demonstrate a 1-h differences, according to our (post hoc) power analysis. Patient and treatment characteristics are shown in Table 1. Female age, body mass index (BMI) and number of aspirated oocytes were comparable between the two groups. Ovarian stimulation using GnRH agonist co-treatment was more common in the Vitrolife group than in the SAGE group (30.0% and 77.1%, respectively, *p* < 0.001). The Vitrolife group included less ICSI treatment cycles than the SAGE group (41.1% and 98.0%, respectively, *p* < 0.001).

**Table 1:** Baseline characteristics of study population

	Vitrolife G-1 PLUS		SAGE 1-Step		
	Median/n	IQR / %	Median/n	IQR / %	*P*-value
Women (embryos)	253 (671)		148 (517)		
Age, years	34.5	30.6–38.8	34.0	29.8–38.1	0.458
BMI, kg/m^2^*	23.4	21.3–26.8	24.0	21.8–26.4	0.233
Fertilisation, ICSI	104	41.1	145	98.0	< 0.001
Stimulation protocol, agonist**	27	30.0	108	77.1	< 0.001
Oocytes aspirated per patient, n	7	4–10	6	4–9	0.572

BMI, body mass index. ICSI, intracytoplasmic sperm injection. IQR, interquartile range

^*^ missing n = 201. ** missing n = 171

### Morphokinetic parameters

**Fig. 1:**
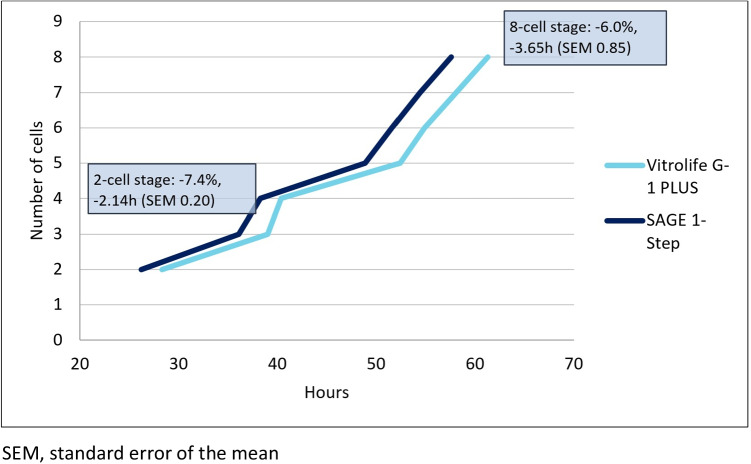
Morphokinetic developmental trajectories of preimplantation embryos cultured in Vitrolife G-1 PLUS (light blue) and SAGE 1-Step medium (dark blue)

After adjustment for clustering, female age, fertilisation method, type of ovarian stimulation co-treatment, oxygen levels at culture and overall improvement in embryo development over time, linear mixed model analysis shows that embryos cultured using SAGE medium reached the pronuclear fading moment 2.13 (95%CI: -3.41, -0.84, *p* = 0.001) hours faster than embryos cultured in Vitrolife medium (Table 2). The cleavage divisions to the 2-, 3-, 4-, 5-, 6-, 7 and 8-cell stage occurred 2.28 (95%CI: -3.66, -0.89, *p* = 0.001), 2.34 (95%CI: -4.00, -0.64, *p* = 0.07), 2.41 (95%CI: -4.11, -0.71, *p* = 0.006), 2.54 (95%CI: -4.90, -0.18, *p* = 0.035), 3.58 (95%CI: -6.08, -1.08, *p* = 0.005), 5.62 (95%CI: -8.80, -2.45, *p* = 0.001) and 5.32 (95%CI: -9.21, -1.42, *p* = 0.008) hours faster, respectively. SAGE embryos reach the 2-cell stage 7.4% faster and the 8-cell stage 6.0% faster than Vitrolife embryos (Fig. 1).

**Table 2:** Differences in morphokinetic parameters of IVF and ICSI embryos cultured in SAGE 1-Step compared to embryos cultured in Vitrolife G-1 PLUS medium

Morphokinetic parameter	Model 1		Model 2*		Missings
	Beta (95% CI), hours	*P*-value	Beta (95% CI), hours	*P*-value	
tPNf	-2.10 (-2.68, -1.51)	< 0.001	-2.13 (-3.41, -0.84)	0.001	31
t2	-2.09 (-2.71, -1.46)	< 0.001	-2.28 (-3.66, -0.89)	0.001	21
t3	-2.98 (-3.74, -2.22)	< 0.001	-2.34 (-4.00, -0.64)	0.007	34
t4	-2.10 (-2.86, -1.34)	< 0.001	-2.41 (-4.11, -0.71)	0.006	50
t5	-3.39 (-4.43, -2.34)	< 0.001	-2.54 (-4.90, -0.18)	0.035	97
t6	-3.12 (-4.29, -1.94)	< 0.001	-3.58 (-6.08, -1.08)	0.005	452
t7	-3.43 (-4.92, -1.95)	< 0.001	-5.62 (-8.80, -2.45)	0.001	550
t8	-3.27 (-5.17, -1.38)	0.001	-5.32 (-9.21, -1.42)	0.008	626
t3-t2	-0.82 (-1.36, -0.28)	0.003	0.18 (-1.03, 1.39)	0.767	34
t5-t4	-1.49 (-2.25, -0.73)	< 0.001	-0.18 (-1.88, 1.53)	0.839	97
t3-tPNf	-0.93 (-1.48, -0.39)	0.001	-0.15 (-1.37, 1.07)	0.807	63
(t5-t3)/(t5-t2)	0.00 (-0.02, 0.03)	0.716	-0.03 (-0.09, 0.02)	0.205	97

^*^Adjustments were made for female age, fertilisation method, type of ovarian stimulation, lowered oxygen culture and overall improvement in embryo development over time. CI, confidence interval

The SAGE group contained more ICSI treatment cycles, and to investigate the possibility that this is the underlying cause for the faster developmental kinetics observed in this group, we also performed a sub-analyses of ICSI embryos only (*n* = 757). These analyses showed that ICSI embryos cultured in SAGE (*n* = 513) reach the pronuclear fading moment faster than ICSI embryos cultured in Vitrolife medium (*n* = 244). These analyses confirmed that ICSI embryos cultured in SAGE reach the 2- (-3.09 h (95%CI -5.14, -1.03) *p* = 0.003), 5- (-4.67 h (95%CI -9.47, -0.05) *p* = 0.047), 6- (-7.25 h (95%CI -12.96, -1.55) *p* = 0.013) and 7-cell stage (-9.21 h (95%CI -17.75, -0.67) *p* = 0.035) faster than ICSI embryos cultured in Vitrolife (Table 3).

**Table 3:** Differences in morphokinetic parameters of ICSI embryos cultured in SAGE 1-Step compared to embryos cultured in Vitrolife G-1 PLUS medium

Morphokinetic parameter	Model 1		Model 2*	
	Beta (95% CI), hours	*P*-value	Beta (95% CI), hours	*P*-value
tPNf	-1.91 (-2.60, -1.21)	< 0.001	-2.69 (-4.73, -0.65)	0.010
t2	-1.93 (-2.67, -1.18)	< 0.001	-3.09 (-5.14, -1.03)	0.003
t3	-2.95 (-3.96, -1.94)	< 0.001	-2.22 (-5.58, 1.13)	0.193
t4	-2.19 (-3.21, -1.17)	< 0.001	-1.63 (-5.13, 1.87)	0.360
t5	-2.28 (-4.14, -1.41)	< 0.001	-4.76 (-9.47, -0.05)	0.047
t6	-3.34 (-4.77, -1.90)	< 0.001	-7.25 (-12.96, -1.55)	0.013
t7	-3.86 (-5.62, -2.10)	< 0.001	-9.21 (-17.75, -0.67)	0.035
t8	-4.65 (-6.90, -2.39)	< 0.001	-9.13 (-19.30, 1.05)	0.078
t3-t2	-0.81 (-1.57, -0.05)	0.037	1.13 (-1.52, 3.79)	0.400
t5-t4	-0.70 (-1.66, 0.25)	0.148	-2.92 (-6.44, 0.60)	0.104
t3-tPNf	-1.07 (-1.85, -0.29)	0.008	0.38 (-2.38, 3.14)	0.786
(t5-t3)/(t5-t2)	0.01 (-0.02, 0.05)	0.472	-0.09 (-0.21, 0.04)	0.178

^*^Adjustments were made for female age, type of ovarian stimulation, lowered oxygen culture and overall improvement in embryo development over time. CI, confidence interval

DUC was more prevalent in SAGE embryos than in Vitrolife embryos (*n* = 79 (15.4%) vs. *n* = 57 (8.9%) respectively, *p* = 0.001). Exclusion of DUC embryos did not change the observation of faster embryo development in the SAGE group (data not shown). It is likely that the observation of significantly more DUC embryos in SAGE medium is related to the difference in IVF-to-ICSI treatment ratio between the two culture media, as comparable DUC rates are observed in ICSI embryos only (SAGE: 15.5%; Vitrolife: 12.2%, *p* = 0.235).

To investigate whether the faster development in SAGE medium is related to embryo quality, we performed sub-analyses of only IVF and ICSI embryos that successfully implanted after fresh ET (*n* = 143). These also demonstrated that embryos cultured in SAGE reached the moment of pronuclear fading, t2, t4 and t6 faster than embryos cultured in Vitrolife, suggesting faster development in SAGE medium for embryos with similar implantation potential (Supplemental Table 2).

### Retrospective assignment of KIDScores

**Fig. 2:**
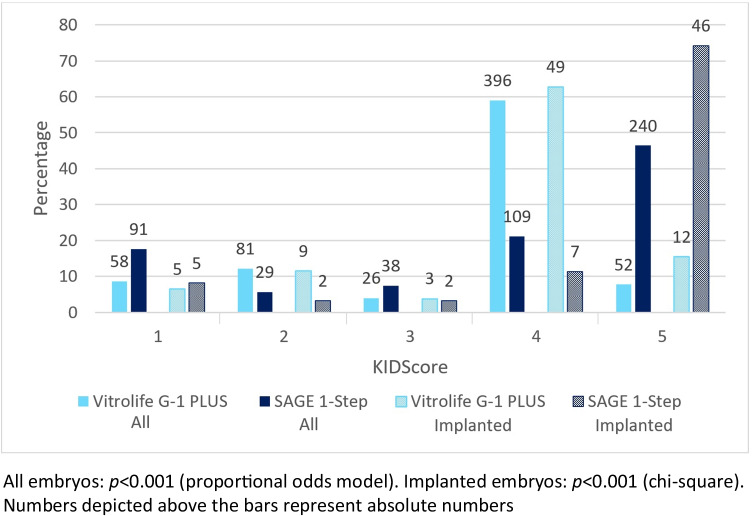
Distribution of KIDScores of embryos cultured in Vitrolife G-1 PLUS (light blue) and SAGE 1-Step medium (dark blue)

The distribution of KIDScores was significantly different between embryos cultured in SAGE and embryos cultured in Vitrolife (proportional odds model: *p* < 0.001) (Fig. 2). Embryos cultured in SAGE fell significantly more often within the optimal time ranges corresponding to a KIDScore of 5 than embryos cultured in Vitrolife (*n* = 240 (46.4%) and *n* = 52 (7.7%), respectively). At the same time, more embryos cultured in SAGE had a KIDScore of 1 than embryos cultured in Vitrolife (*n* = 91 (17.6%) and *n* = 58 (8.6%), respectively). In a small number of embryos, a KIDScore could not be assigned (Vitrolife n = 58 (8.6%), SAGE *n* = 10 (1.9%)) due to the fact that some developmental stages could not be assessed on the available images.

Sub-analysis of the freshly transferred embryos that implanted successfully, demonstrated a similar distribution across the KIDScores (Fig. 2). It was found that the majority of successfully implanted embryos in the Vitrolife group classified for a KIDScore of 4, whereas for successfully implanted SAGE embryos the majority classified for the optimal KIDScore of 5.

### Clinical treatment outcomes

Table 3 demonstrates the clinical outcomes per patient after culture in SAGE and Vitrolife. After fresh ET (*n* = 372), 80 (33.6%) women in the Vitrolife group and 63 (47.0%) women in the SAGE group had a positive hCG test. Crude logistic regression analysis, with Vitrolife as reference, showed an odds ratio (OR) of 1.81 (95%CI: 1.21, 2.71, *p* = 0.004) for positive hCG test, 1.93 (95%CI: 1.29, 2.88, *p* = 0.001) for the presence of a gestational sac, 1.75 (95%CI: 1.14, 2.69, *p* = 0.010) for the presence of foetal heartbeat and 1.45 (95%CI: 0.92, 2.28, *p* = 0.110) for live birth for embryos cultured in SAGE (Table 4). However, after adjustment for female age, fertilisation method and type of ovarian stimulation, none of the clinical outcomes after fresh ET were significantly different.

**Table 4:** Clinical outcomes per patient after culture in SAGE 1-Step compared to culture in Vitrolife G-1 PLUS

	Vitrolife G-1 PLUS	SAGE 1-Step	Crude		Adjusted*		
	*N* (%)	*N* (%)	OR (95% CI)	*P*-value	OR (95% CI)	*P*-value	Missings
After fresh ET	*N* = 238	*N* = 134					
Positive hCG test	80 (33.6)	63 (47.0)	1.81 (1.21, 2.71)	0.004	1.74 (0.82, 3.69)	0.150	0
Gestational sac	76 (31.9)	62 (46.3)	1.93 (1.29, 2.88)	0.001	1.80 (0.85, 3.85)	0.128	0
Foetal heartbeat	61 (25.6)	48 (44.0)	1.75 (1.14, 2.69)	0.010	1.58 (0.71, 3.52)	0.256	0
Live born	54 (23.2)	37 (28.7)	1.45 (0.92, 2.28)	0.110	0.99 (0.43, 2.31)	0.986	10
After fresh and frozen ETs	*N* = 253	*N* = 148					
Cumulative ongoing pregnancy	86 (34.0)	69 (46.6)	1.70 (1.12, 2.57)	0.013	1.80 (0.84, 3.90)	0.134	0

^*^Adjustments were made for female age, fertilisation method and type of ovarian stimulation

ET, embryo transfer. OR, odds ratio. CI, confidence interval

The crude OR for cumulative pregnancy rate, i.e. a pregnancy resulting either after fresh ET or after frozen–thawed ET within a two-year follow-up period after oocyte retrieval, is 1.70 (95%CI 1.12, 2.57, *p* = 0.013) for embryos cultured in SAGE. However, after adjustments, we found no differences for this clinical treatment outcome between the culture media.

## Discussion

The aim of this study was to compare embryo morphokinetics and clinical outcomes between two different embryo culture media used during conventional IVF and during ICSI treatment. We observed that embryos cultured in SAGE culture medium reached the pronuclear fading moment earlier and proceeded faster through all the cleavage divisions than embryos cultured in Vitrolife, regardless of the fertilisation method. These embryos also qualified significantly more often for the highest KIDScore, suggesting a higher implantation potential. However, when only successfully implanted embryos were considered, those cultured in SAGE still showed faster development. Also, we found no significant differences in clinical outcomes, such as implantation and cumulative pregnancy rate, after correction for confounders. This suggests that culture medium has a direct impact on embryo developmental kinetics, but this does not necessarily reflect in implantation potential.

Although the timing of cleavage divisions is different between embryos cultured in Vitrolife and embryos cultured in SAGE, it remains unknown which specific components drive these differences. It has been suggested that variations in pyruvate, lactate, amino acid and glucose concentrations between culture media may affect embryo morphokinetics and metabolism [43–45]. In mice, it has been shown that fast-cleaving embryos have a higher glucose consumption rate than slow-cleaving embryos [46]. This is seemingly in contrast to our results, as we observe faster development of embryos cultured in the medium with lower glucose content (SAGE). Yet, during the first stages of human preimplantation embryo development pyruvate and lactate are the primary source of energy, and high levels of glucose during the cleavage stages might be counterproductive for preimplantation development [47, 48].

Other components in culture media, such as amino acid concentrations, are also likely to have a significant effect on early embryo development. Amino acids are important during the cleavage stages, as they regulate embryo physiology [49–52]. It has been demonstrated that embryos with a lower amino acid turnover develop more often to the blastocyst stage and have less DNA damage [53–55]. This is in line with the ‘quiet embryo hypothesis’ of Leese [56], which postulates that embryos with a low metabolism have a higher viability as they endure environmental stress relatively well. However, the presence of amino acids in culture medium is ambivalent, as they also contribute to ammonium buildup. High levels of ammonium can have a detrimental effect on embryo development and pregnancy rates [57, 58]. Minimising ammonium buildup is especially critical for single-step media, as not refreshing the media might otherwise result in detrimental ammonium levels during 5 days of culture.

In addition to the unknown mechanisms underlying the differences in morphokinetics, the clinical impact of these differences is also largely unknown. Although several studies indicate that fast-developing embryos have a higher potential for implantation, the added clinical value of numerous (de)selection tools has yet to be conclusively established [26, 46, 59–64]. For example, after undisturbed culture in a self-contained incubator, clinical outcomes are comparable between morphokinetic-based and morphology-based embryo selection [61]. It is likely that the differences we observed in individual morphokinetic parameters between culture media, translated to differences in our studied deselection tool, the KIDScore. We showed that a larger proportion of SAGE embryos qualified for the highest score, compared to Vitrolife embryos, which might be explained by more embryos reaching the 8-cell stage before 66 h after fertilisation. Although the predicted implantation potential, i.e. the KIDScore, was higher in the SAGE group, the observed implantation potential, as well as cumulative pregnancy rate, were comparable between the two culture media. Interestingly, after implantation, SAGE embryos also exhibit faster growth and development than Vitrolife embryos [21].

Since the KIDScore was developed for general applicability in different treatment and culture conditions, its test characteristics were not studied in specific culture media. Yet, our results suggest that the type of culture medium may impact the discriminatory value of the KIDScore. For example, even in embryos that implanted successfully, the KIDScore was significantly different between culture media. Moreover, the KIDScore distribution of transferred and cryopreserved Vitrolife embryos is considerably different from the distribution described by Petersen et al. [26]. However, it should be noted that the KIDScore algorithm and its distribution are based on embryos that had been selected for transfer, whereas we included all embryos that had been selected for transfer and cryopreservation.

Since we show that type of culture medium affects multiple individual morphokinetic parameters, our findings may have consequences for the discriminatory value and applicability of other (de)selection tools. Therefore, it is recommended that IVF clinics should either validate morphokinetic-based selection tools prior to implementation or develop their own clinic-specific selection tool, since the ideal developmental kinetics of human embryos under different culture conditions remains largely unknown.

Previous research regarding the effect of culture medium on morphokinetic parameters in human embryos is scarce and reports conflicting results. Some studies report no effect of culture medium on morphokinetic parameters, whereas others demonstrate faster development for single-step culture medium [28–31]. Despite the fact that randomisation was applied in all of these studies, sample sizes were small, statistical analyses were mainly based on t testing and none of the studies corrected for clustering of multiple embryos from one woman. Without considering clustering, it is possible that observed effects are essentially based on a large number of embryos from only a few patients. Therefore, we applied linear mixed models, which enable precise modelling of embryo development by taking into account both clustering as well as confounders such as age and type of ovarian stimulation. Also, a post hoc power analysis confirmed that the size of our study population is sufficient to demonstrate a 1-h difference in preimplantation development. Another benefit of this patient registry cohort is the diverse study population. Our study population consisted of IVF as well as ICSI treatments, and different ovarian stimulation approaches have been used, which increases the generalisability of our results. However, the ratio of IVF to ICSI treatments was not comparable within the groups of studied culture media. It is essential to correct for fertilisation method when studying time-lapse parameters, as differences between IVF and ICSI embryos have been suggested [65]. We approached this in two ways: We adjusted for fertilisation method in our linear mixed model and also performed a sensitivity analysis in ICSI embryos only. By doing so, we demonstrate an impact of the culture medium as early as the moment of pronuclear fading in analyses both including all embryos as well as including ICSI embryos only. However, others have suggested to use tPNf as a starting point [41]. With this method, a potential impact on the moment of pronuclear fading is missed, and observations in subsequent cleavage stages might also be affected.

The main limitation of our study is that we only data have data available from the cleavage stage development and not up to the blastocyst stage. As blastocyst transfer is associated with higher pregnancy and live birth rates [66], assessment of this stage is likely to be more informative. This study was performed in a time in which fresh transfer of cleavage embryos was routine care in most IVF clinics, including ours. Future research should include blastocyst development as it may provide a more extensive understanding of the impact of culture medium. Although our patient data for BMI and stimulation regimen is incomplete, the sensitivity analysis showed similar results for patients with complete data (*n* = 230) and patients with incomplete data (*n* = 171), suggesting a low risk of selection bias (data not shown). The studied culture media were used during consecutive time periods. To minimise this effect of confounding due to a secular trend, we also corrected for laboratory-specific changes and the moment of culture. Finally, despite the adjustments for potential confounders in our analyses, residual confounding cannot be excluded, as this is inherent to the observational study design.

## Conclusion

Our results demonstrate an impact of culture medium on preimplantation embryo developmental kinetics and this affects classification within the KIDScore algorithm. Other studies show that culture medium can alter post-implantation growth as early as the second trimester of pregnancy and even impact offspring health at nine years of age [18, 67]. However, it remains unknown which specific component(s) in culture medium drive these differences. Since over half a million babies are born after IVF and ICSI treatment each year, more studies should shift the focus from the effects of IVF culture conditions on implantation to their effect on (preimplantation) embryo development [68]. Moreover, early embryonic development has been linked to postnatal health and development [69]. This, in combination with the results of Dumoulin et al., further warrants the need to elucidate the association between preimplantation embryo kinetics, post-implantation growth and offspring health.

## Supplementary Information

Below is the link to the electronic supplementary material.

Supplementary file1 (DOCX 17 KB)

## Data Availability

The data underlying this article cannot be shared publicly due to the privacy of individuals that participated in the study.

## Code Availability

The code underlying this article can be shared on reasonable request to the corresponding author.
