# Supplementary material for: The Impact of Culture Medium on Morphokinetics of Cleavage Stage Embryos: An Observational Study
Source: Reprod Sci. 2022 May 9;29(8):2179–89. doi: 10.1007/s43032-022-00962-7 (PMC9352745; doi:10.1007/s43032-022-00962-7)
Supplement: Supplementary file 1 — Supplementary file1 (DOCX 17 KB) [file 43032_2022_962_MOESM1_ESM.docx]

**SUPPLEMENTAL MATERIAL**

**Supplemental table 1.** Morpokinetic parameters of the KIDScore algorithm.

| **Morphokinetic parameter** |
| --- |
| Number pronuclei equals 2 at the 1-cell stage |
| Time from insemination to pronuclear fading (tPNf) |
| Time from insemination to the 2-cell stage (t2) |
| Time from insemination to the 3-cell stage (t3) |
| Time from insemination to the 5-cell stage (t5) |
| Number of cells 66 hours after fertilisation |

**Supplemental table 2.** Differences in morphokinetic parameters of implanted embryos (n=143) cultured in SAGE 1-Step compared to Vitrolife G-1 PLUS culture medium after fresh embryo transfer.

|  | **Model 1** |  | **Model 2*** |  | **Missings** |
| --- | --- | --- | --- | --- | --- |
| **Morphokinetic parameter** | **Beta (95% CI), hours** | ***P-*value** | **Beta (95% CI), hours** | ***P-*value** |  |
| tPNf | -2.51 (-3.38, -1.64) | <0.001 | -2.44 (-4.42, -0.45) | 0.017 | 1 |
| t2 | -2.65 (-3.62, -1.68) | <0.001 | -2.80 (-4.99, -0.61) | 0.013 | 0 |
| t3 | -2.79 (-4.06, -1.52) | <0.001 | -2.66 (-5.50, 0.18) | 0.066 | 2 |
| t4 | -2.82 (-3.98, -1.65) | <0.001 | -3.45 (-6.01, -0.80) | 0.011 | 2 |
| t5 | -2.88 (-4.71, -1.04) | 0.002 | -3.70 (-7.99, 0.59) | 0.090 | 5 |
| t6 | -4.12 (-6.04, -2.21) | <0.001 | -4.34 (-8.53, -0.14) | 0.043 | 45 |
| t7 | -3.78 (-6.03, -1.53) | 0.001 | -3.81 (-8.81, 1.78) | 0.133 | 58 |
| t8 | -2.75 (-5.29, -0.21) | 0.034 | -2.46 (-7.91, 3.00) | 0.372 | 75 |
| t3-t2 | -0.04 (-1.07, 0.98) | 0.935 | 0.36 (-1.95, 2.66) | 0.760 | 2 |
| t5-t4 | -0.26 (-1.70, 1.18) | 0.724 | -0.09 (-3.40, 3.23) | 0.959 | 5 |
| t3-tPNf | -0.21 (-1.22, 0.79) | 0.675 | -0.07 (-2.31, 2.17) | 0.950 | 3 |
| (t5-t3)/(t5-t2) | -0.01 (-0.06, 0.04) | 0.651 | -0.05 (-0.15, 0.06) | 0.351 | 5 |

*Adjustments were made for female age, fertilisation method, type of ovarian stimulation, lowered oxygen culture and overall improvement in embryo development over time. CI, confidence interval.
